# Mr. Whately on Aqueous Injection in Hydrocele

**Published:** 1811-08

**Authors:** Thomas Whately

**Affiliations:** Grafton Street


*To the Editors of the Medical and Physical Journal.*

Gentlemen,

For some years past I have used cold water only, injected in the usual manner, as a cure for the hydrocele; though in some cases, within this period, I have used an injection of Port wine and water. Unfortunately, I have not kept a regular account of the number of cases in which the first of these methods of cure was adopted; but to the best of my recollection, they have been from fifteen to twenty. Three of them have occurred within the last three months, and I do not recollect a single instance of the failure of cure where this method has been pursued. In most of the cases, the inflammation and confinement have been less than occurred where the Port wine injection was used. On the ground of experience, therefore, I can venture to recommend this method to the attention of other practitioners.

If you think this little communication worthy a place in your valuable miscellany, it is at your service.

I am, Gentlemen,

Your obedient humble Servant,

THOMAS WHATELY.

*Grafton Street, May* 29, 1811.

